# Mapping the genetic basis of diabetes mellitus in the Australian Burmese cat (*Felis catus*)

**DOI:** 10.1038/s41598-020-76166-3

**Published:** 2020-11-05

**Authors:** Georgina Samaha, Claire M. Wade, Julia Beatty, Leslie A. Lyons, Linda M. Fleeman, Bianca Haase

**Affiliations:** 1 Faculty of Science, Sydney School of Veterinary Science, University of Sydney, Sydney, NSW Australia; 2 School of Life and Environmental Sciences, The University of Sydney, Sydney, NSW Australia; 3 Department of Infectious Diseases and Public Health, City University of Hong Kong, Kowloon, Hong Kong SAR People’s Republic of China; 4 Department of Veterinary Medicine and Surgery, College of Veterinary Medicine, University of Missouri, Columbia, MO USA; 5 Animal Diabetes Australia, Melbourne, VIC Australia

**Keywords:** Animal breeding, Genetic association study, Genetic linkage study, Haplotypes, Medical genetics

## Abstract

Diabetes mellitus, a common endocrinopathy affecting domestic cats, shares many clinical and pathologic features with type 2 diabetes in humans. In Australia and Europe, diabetes mellitus is almost four times more common among Burmese cats than in other breeds. As a genetically isolated population, the diabetic Australian Burmese cat provides a spontaneous genetic model for studying diabetes mellitus in humans. Studying complex diseases in pedigreed breeds facilitates tighter control of confounding factors including population stratification, allelic frequencies and environmental heterogeneity. We used the feline SNV array and whole genome sequence data to undertake a genome wide-association study and runs of homozygosity analysis, of a case–control cohort of Australian and European Burmese cats. Our results identified diabetes-associated haplotypes across chromosomes A3, B1 and E1 and selective sweeps across the Burmese breed on chromosomes B1, B3, D1 and D4. The locus on chromosome B1, common to both analyses, revealed coding and splice region variants in candidate genes, *ANK1, EPHX2* and *LOX2,* implicated in diabetes mellitus and lipid dysregulation. Mapping this condition in Burmese cats has revealed a polygenic spectrum, implicating loci linked to pancreatic beta cell dysfunction, lipid dysregulation and insulin resistance in the pathogenesis of diabetes mellitus in the Burmese cat.

## Introduction

Domestic cats (*Felis catus*), a common household pet share with humans many environmental and lifestyle risk factors for metabolic disorders like diabetes mellitus. Feline diabetes mellitus (FDM; OMIA 000277-9685) affects approximately one in 200 cats. Similarities in clinical presentation, pathological findings and risk factors between FDM and human type 2 diabetes (T2D) are well-established^1–4^. Both are characterised by inadequate insulin secretion resulting in absolute or partial insulin deficiency and varying degrees of insulin resistance in peripheral tissues. The prevalence of FDM has been increasing over recent decades, with obesity, diet, over-nutrition and lack of physical inactivity all implicated as risk factors^5,6^. In humans, T2D is known to have a complex underlying genetic architecture, with many genetic risk loci identified by genome wide association studies (GWAS). Such studies have consistently identified T2D associated loci containing genes that alter glucose metabolism or pancreatic β-cell function^7,8^.

Among recognised cat breeds, the incidence of FDM is highest in Burmese cats^9,10^. In Australia, Britain and Europe, Burmese cats are approximately four times more likely to develop FDM than domestic cats of other breeds or breed mixes^11^. In contrast, the same heightened risk has not been observed in the North American Burmese population which is regarded as genetically distinct^12–14^. Companion animals are increasingly used to model human diseases^15,16^. Studying the basis of diabetes mellitus in the naturally occurring Burmese model circumvents the drawbacks associated with in vivo experimental work involving induced illness in healthy animals and transgenic animals. This includes welfare and the inability to adequately model the disease state^17^. Compared with humans, the relatively short lifespan of cats permits observation of the natural progression of an illness over a compressed timeline.

Breed-specific phenotypes in cats result from selective breeding for desired characteristics such as coat colour and texture, body size and morphology. Breed-specific traits are often accompanied by breed-specific susceptibility to genetic diseases compared with outbred populations. Heritable diseases disproportionately affecting Burmese cats include craniofacial defect^18^, hypokalaemia^19^, hyperlipidaemia^20^, orofacial pain syndrome^21^ and diabetes^22^. Regarding genetic diversity, the Burmese breed has among the lowest levels of heterozygosity, most extensive linkage disequilibrium (LD) and highest inbreeding coefficients in recognised cat breeds^14,23^. This simplified genetic architecture is beneficial for mapping the genetic basis of breed-specific traits. Extensive LD reduces the number of markers required to tag segregating haplotypes^23,24^, making Burmese suitable for analysis on the Illumina Infinium iSelect 63 k Cat DNA genotyping array that is used in this study.

The closed breeding systems used to maintain pedigreed breed lines leave distinct genomic traces, particularly when population bottlenecks, such as the importation of individuals to new countries, create founder effects. The extent and structure of LD varies widely across chromosomes and regions of long-range LD can indicate partial or complete selective sweeps of functional significance in a population. Across species, the process of intensive selection for breed-specific traits influences the frequency, distribution and length of runs of homozygosity (ROH)^25^. ROH exist as long tracts of homozygous genotypes and are often the result of consanguineous matings, but they can arise by other mechanisms^25–27^. Detecting ROH can identify genes that have been subjected to selection in population genetic analyses^28,29^. For example, ROH detection has been used to map causative recessive variants segregating within human families and canine breeds in complex and Mendelian diseases^30–33^. Enrichment of deleterious variants surrounding regions of selection in domesticated breeds suggest deleterious alleles may ‘hitchhike’ with nearby positively selected alleles, supporting a link between disease heritability and breed-specific traits^33–35^.

The unique demographic history of the Burmese breed is characterised by reduced effective population sizes, potential founder effects and genetic bottlenecks that may account for differential enrichment of FDM between different breeding populations. In a population with an already reduced genetic diversity, we predict GWAS and ROH analyses offer an opportunity to detect FDM-risk loci segregating in the Australian Burmese breeding population. We used genotyping array and whole genome sequence data to, (1) conduct case–control GWAS of diabetic and non-diabetic Burmese cats, (2) Identify FDM-associated risk haplotypes and (3) perform genome-wide characterisation of Burmese ROH to expose risk loci that differentiate the heightened prevalence of FDM in this breed.

## Results

### Remapping array variants to feline 9.0 genome assembly

As inconsistencies between genome assemblies can complicate GWAS, the SNV locations of array variants were updated to the most recent feline reference assembly (felCat9). Original locations were included in a manifest provided with the Illumina iSelect 63 Cat DNA genotyping array. For the 62,897 variants on the feline genotyping array, 61,371 (97.6%) were remapped. This comprised 58,674 autosomal variants and 2697 markers on chromosome X. The marker counts and statistics per chromosome (Table S1a) and the updated map file (Table S1b) are presented in Supplementary data. The largest gap between consecutive markers spanned 82.3 Mb and was detected on chromosome E1. The average distance between markers was 39.7 kb. As expected, gaps and low-density distributions of SNVs were consistently observed flanking centromeres for each chromosome.

### Genome-wide and haplotype association analyses

**Figure 1:**
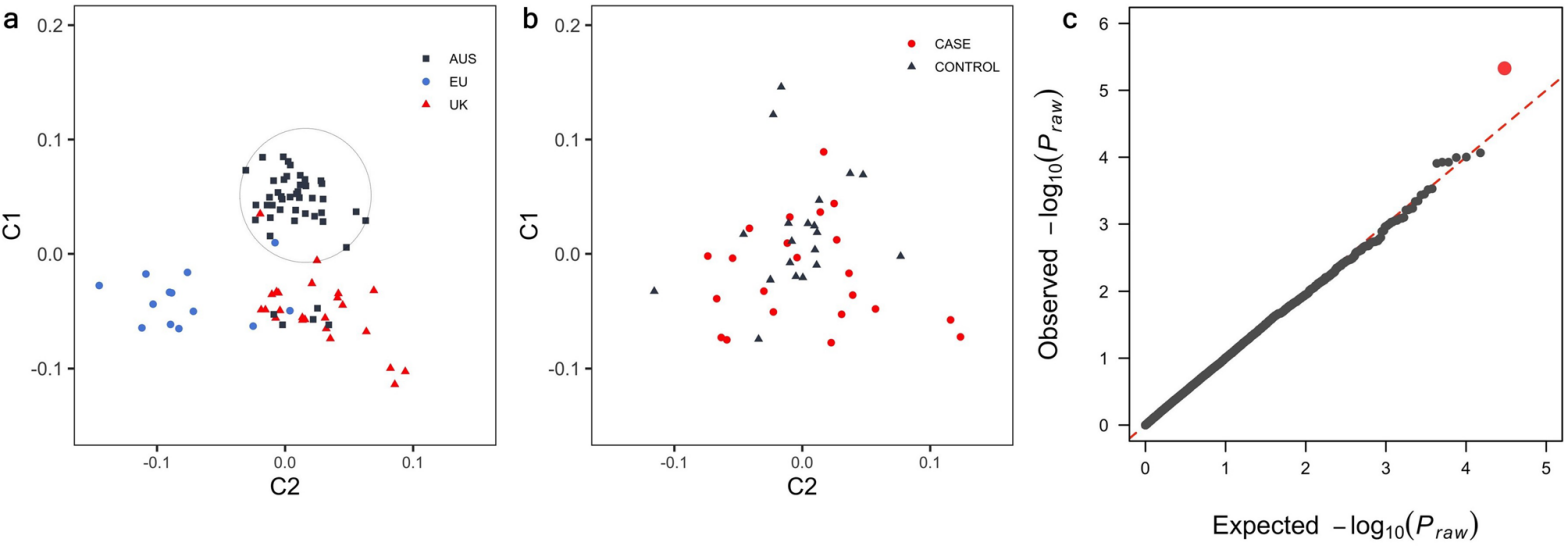
Multi-dimensional scaling and quantile–quantile plot of diabetic and non-diabetic Burmese cats. (**a**) Multi-dimensional scaling distribution of Burmese cats of Australian, British and European (HK) provenance in two dimensions. Samples included within the Australian cluster (circled) were included in the initial case–control association analysis. (**b**) Multi-dimensional scaling distribution of diabetic and non-diabetic Burmese cats in the Australian cluster shown in two dimensions (**c**) Quantile–quantile plot showing limited inflation of the test statistics with a genomic inflation factor (λ = 1.03).

**Figure 2:**
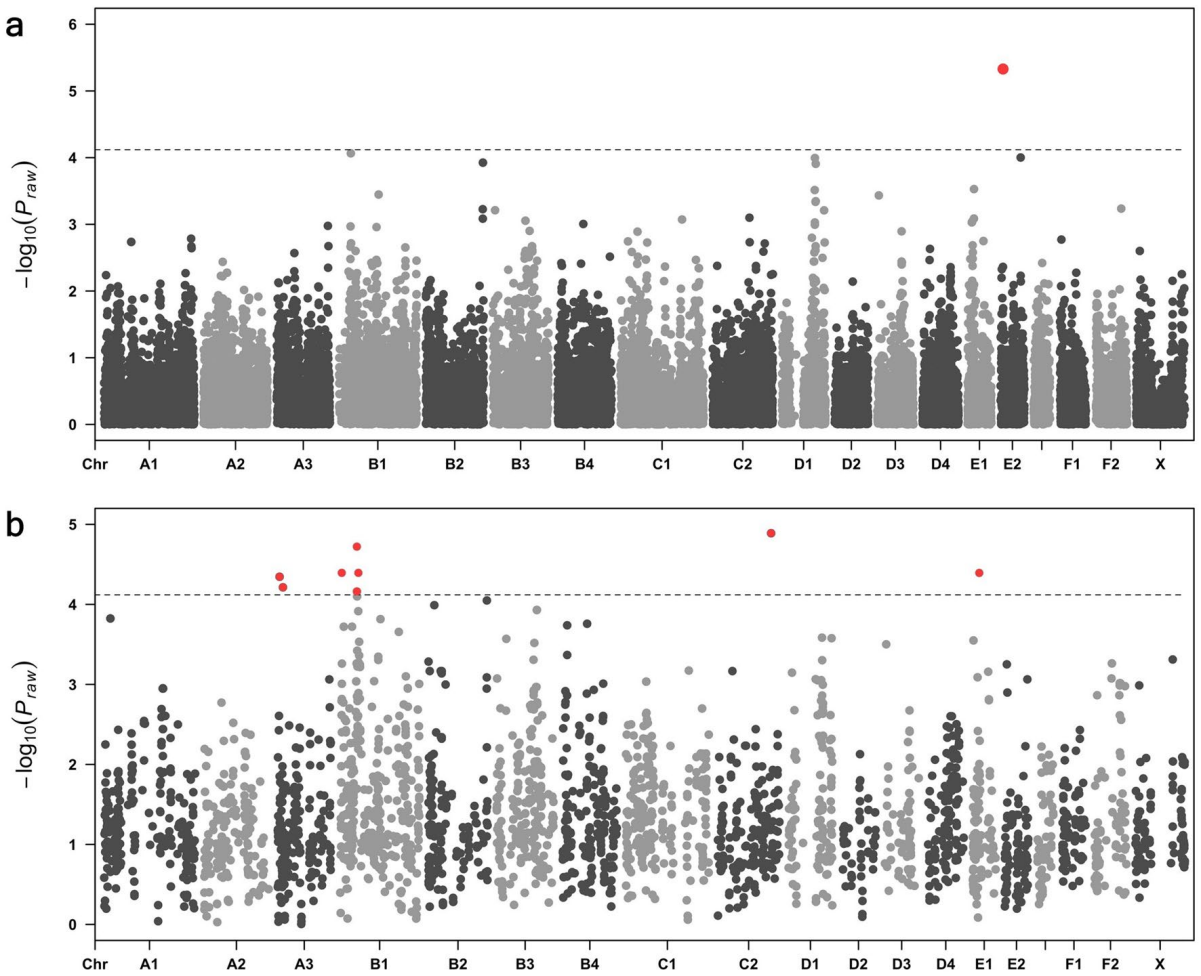
Case–control genome wide association analysis performed in two stages in the Burmese breed. (**a**) Manhattan plot summarising initial analysis of 20 cases and 22 controls in the Australian cluster shows a marker on chromosome E1 b. The top 10% of markers (3022 SNVs) run in the initial association analysis were run in an expanded association analysis of 82 Burmese cats of Australian, European and British provenance, comprising 22 cases and 60 controls. Signals that maintained or increased their significance above the empirical genome-wise significance threshold (*P* < 7.6 × 10^−5^) are highlighted in red. Loci passing this threshold were observed on chromosomes A3, B1 and E1.

Samples from eighty-two Burmese cats that were genotyped on the Illumina Infinium Feline 63 k iSelect DNA array passed quality control. Multidimensional scaling (MDS) revealed some geographical clustering of cat populations (Fig. 1a). Cats from Europe (EU) clustered distinctly from Australian (AUS) and British (UK) samples. Given geographical clustering of cases, the GWAS was first run in Australian cats only to control for population stratification (analysis 1). Analysis 1 comprised of 22 cases and 20 controls, Cases and controls within the cohort of samples used in the initial case–control GWAS (analysis 1) which were evenly dispersed across the population cohort (herein referred to as the ‘Australian cluster’) (Fig. 1b). For analysis 1, the QQ-plot revealed a deviation from the expected *P*-value distribution only in the tail (λ = 1.03) (Fig. 1c). This analysis totalled 30,212 SNVs and one marker at E2:6,883,182 (P_raw_ = 4.68 × 10^−6^) passed the empirical genome-wide significance threshold (Fig. 2a).

The 3021 SNVs that comprised the top 10% associated signals from analysis 1 were run in an expanded cohort of Australian, European and British Burmese cats (22 cases, 60 controls) (analysis 2). SNVs passing genome-wide significance from analysis 2 were observed on chromosomes A3, B1, C2 and E1 (Fig. 2b). The most significantly associated marker from analysis 2 was C2: 139,466,787 (P_genome_ = 1.26 × 10^−5^). This SNV was found not to be in LD with any surrounding markers and was excluded from further analysis. Additional SNVs passing genome-wide significance on chromosomes A3, B1 and E1 were observed within intronic regions of Guanine nucleotide-binding protein G subunit *(GNAS) (*A3:4,665,802; P_genome_ = 4.44 × 10^−05^), Growth regulating oestrogen receptor binding 1 (*GREB1) (*A3:134,518,080; P_genome_ = 6.13 × 10^−05^), Zinc finger matrin-type 4 (*ZMAT4) (*B1:44,235,566; P_genome_ = 6.85 × 10^−05^) and EF-hand calcium binding domain 5 *(EFCAB5) (*E1:16,917,823; P_genome_ = 4.02 × 10^−05^) genes.

Five regions of association on chromosomes A3, B1 and E1 were defined based on LD clumping with r^2^ > 0.8 to the top regional SNV and used to identify FDM-associated haplotypes within each of these clumps (Table 1). The highest associated haplotype spanned the interval chrA3:134,425,431–134,601,739 bp. This region spanned genes; Lipin-1 *(LPIN1),* Neurotensin receptor 2 (*NTSR2)* and *GREB1* and harboured a SNV within *GREB1* (*P* = 4.33 × 10^−06^) unique to cases.

**Table 1:** LD clumping and haplotype analysis of association signals from analysis 2 identified risk haplotypes on chromosomes A3, B1 and E1 across 82 Burmese cats.

Chr	Position	*P*	Allele	Haplotype block	Size (kb)	Associated haplotype	Case:control freq	Haplotype P	Genes
A3	4,665,802	4.44E−05	C	4,396,199–4,741,413	345	GGCG	0.232:0.042	0.0372	*ATP5F1E TUBB1 PRELID3B NELFCD GNAS*
	134,518,080	6.13E−05	A	134,425,431–134,601,739	176	GAAG	0.168:0	7.00E−04	*LPIN1 NTSR2 GREB1*
B1	44,398,531	1.88E−05	C	43,116,211–44,435,725	1319	AGGAGAGAGAGCGAAGGGCA	0.182:0.017	2.50E−04	*ANK1 GINS4 GPAT4 SFRP1 ZMAT4*
	48,213,950	3.98E−05	A	48,056,021–48,848,288	792	AAAGGGCAAGAACGAGA	0.273:0.033	4.69E−06	*UNC5D*
E1	16,917,823	4.02E−05	A	16,577,377–17,262,839	685	GGGGGCAAGAGGAA	0.636:0.898	0.0052	*TMIGD1 BLMH SLC6A4 NSRP1 EFCAB5 SSH2*

The second highest associated haplotype spanned the interval chrB1:48,056,021–48,848,288 bp (*P* = 4.69 × 10^−06^) and was present in 27.3% of cases and 3.3% of controls. Across this region, seven synonymous and splice region variants in Cathepsin Z (*CTSZ),* Negative elongation factor CD (*NELFCD*), Neuroendocrine secretory protein 55 *(NESP55),* Ankyrin 1 *(ANK1*) and UNC-5 netrin receptor *(UNC5D)* (Table 2), 708 intronic, two splice region variants, two 5′ UTR variants in *GPAT4* and *GINS4* and seven 3′ UTR variants in *CTSZ* and *NELFCD* matched risk haplotypes across the five FDM-associated haplotype blocks (Table S3). Most notably, variants matching the FDM-risk haplotype on B1 were identified in common T2D candidate gene, *ANK1*. Of WGS samples, two cases were heterozygous for both *ANK1* splice variants, all other individuals were homozygous for the reference allele. SNV, g.43227755C > T, was located within 5 bp of the region of a constitutive donor splice site at the start of intron 4 and g.43235911C > T was located 2 bp upstream of a constitutive acceptor splice site at the end of intron 9.

**Table 2:** Coding variants matching FDM-risk haplotypes across GWAS loci on chromosomes A3 and B1 and variants segregating in cases across both ROH on B1.

Chromosome	Position (gDNA)	Sequence change (cDNA)	Variant id	Gene	Consequence	Location
A3	g.4531693	c.1803G > A	–	*CTSZ*	Synonymous	Exon 5
	g.4538198	c.811C > T	–	*NELFCD*	Synonymous	Exon 7
	g.4538472	c.751C > T	rs783705891	*NELFCD*	Synonymous	Exon 6
	g.4678284	c.528A > G	–	*NESP55*	Synonymous	Exon 1
	g.4678485	c.729G > A	–	*NESP55*	Synonymous	Exon 1
B1	g.35028512	c.474G > A	–	*LOXL2*	Missense	Exon 2
	g.35028658	c.620C > T	–	*LOXL2*	Synonymous	Exon 2
	g.35028712	c.674G > A	–	*LOXL2*	Synonymous	Exon 2
	g.35028781	c.743C > T	–	*LOXL2*	Synonymous	Exon 2
	g.35274559	c.136G > A	–	*PEBP4*	Missense	Exon 1
	g.43228852	c.768C > T	–	*ANK1*	Synonymous	Exon 5
	g.43227755C > T	-	–	*ANK1*	Splice region	Intron 4
	g.43235911C > T	-	–	*ANK1*	Splice region	Intron 9
	g.48700205	c.1176G > A	rs783910583	*UNC5D*	Synonymous	Exon 10

### Runs of homozygosity

**Figure 3:**
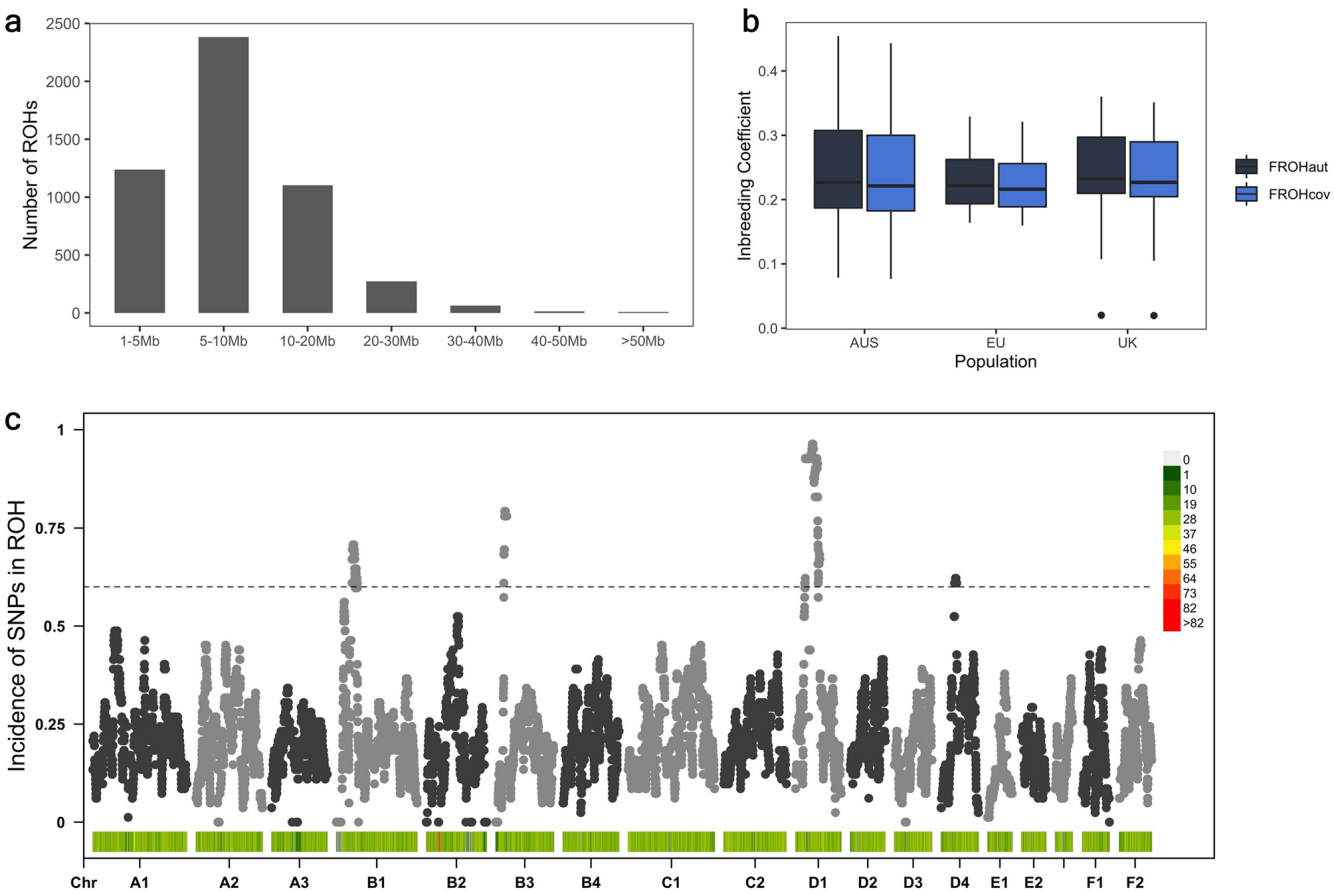
ROH analyses on 82 Burmese samples of Australian, British and European provenance. (**a**) Most ROH were found in the size ranges 1–20 Mb across all individuals. (**b**) Inbreeding coefficient measurements were equivalent across all populations. c. Autosomal distribution of the incidence of SNVs in ROH measured across all samples.

The total number of SNVs included in ROH analysis was 52,933. A total of 5079 ROH were identified across all individuals. The distribution of sizes of ROH was consistent across populations. Most of the observed ROH were less than 20 Mb in size, with 46.9%, 48.1% and 46.5% being observed between 5 and 10 Mb in size (Fig. 3a). Limited by the distribution and density of SNVs on the feline genotyping array, no ROH < 1 Mb were detected. Australian samples displayed larger spans of ROH, with two samples displaying a total length of ROH greater than 750 Mb. These two samples displayed the highest inbreeding coefficients. No correlation was observed between case–control status and the total length of ROH, number of ROH or extent of inbreeding in Burmese cats. Inbreeding coefficient measures FROH_cov_ and FROH_aut_ across all samples were highly correlated (Fig. 3b; r = 1.0; *P* < 2.26 × 10^−16^). While FROH_aut_ is the more commonly reported inbreeding coefficient statistic, FROH_cov_ allows maximal possible genome coverage. Hence, we have reported both here. FROH_aut_ and FROH_cov_ reported an inbreeding coefficient ~ 0.24 across all samples. Inbreeding (FROH_cov_) was consistent among Australian (FROH_cov_ = 0.25), UK (FROH_cov_ = 0.23) and EU (FROH_cov_ = 0.22) populations (Fig. 3b).

Genomic regions containing the most common ROH across all samples were identified across all autosomes (Fig. 3c). ROH on chromosomes B1, B3, D1 and D4 revealed regions potentially under selection in the breed, with both ROH on chromosome B1 containing FDM-risk variants (Table 2). ROH spanning chrB3:20,948,939–28,536,357 contained 22 genes, none of which are associated with known feline phenotypes. This ROH was observed in > 75% of samples and was syntenic with a region of human chromosome 15, previously implicated in Prader Willi syndrome (PWS) (OMIM 176270) and oculocutaneous albinism (OMIM 203200). FDM-risk variants were found to segregate in Non-Imprinted gene in Prader-Willi syndrome/Angelman syndrome (*NIPA1;* OMIM:608145) and Gamma-aminobutyric Acid Receptor, Gamma-3 (*GABRG3;* OMIM:600233).

An ROH spanning chrD1:37,995,037–57,240,116 upstream of the FDM-associated locus was observed in > 90% of all Burmese cats. This locus contained the *Tyrosinase* (*TYR*) gene that has been previously Intronic risk variants in glutamate receptor, metabotropic 5 (*GRM5)*, Dexamethosone-induced gene-2 (*DIG2)* and Myosin VIIA (*MYO7A)* gene were found across WGS samples. The FDM locus with indicative association on chromosome D1 was 40 Mb upstream of the ROH spanning chrD1:37,995,037–57,240,116. The ROH on chrD4:34,892,052–40,360,453, observed in 62% of Burmese samples, spanned the Tyrosinase-related protein 1 (*TYRP1*) gene, implicated in brown coat colour in cats (OMIA 001249-9685). Intronic risk variants in protein tyrosine phosphate receptor delta (*PTPRD*) were detected in WGS samples.

**Figure 4:**
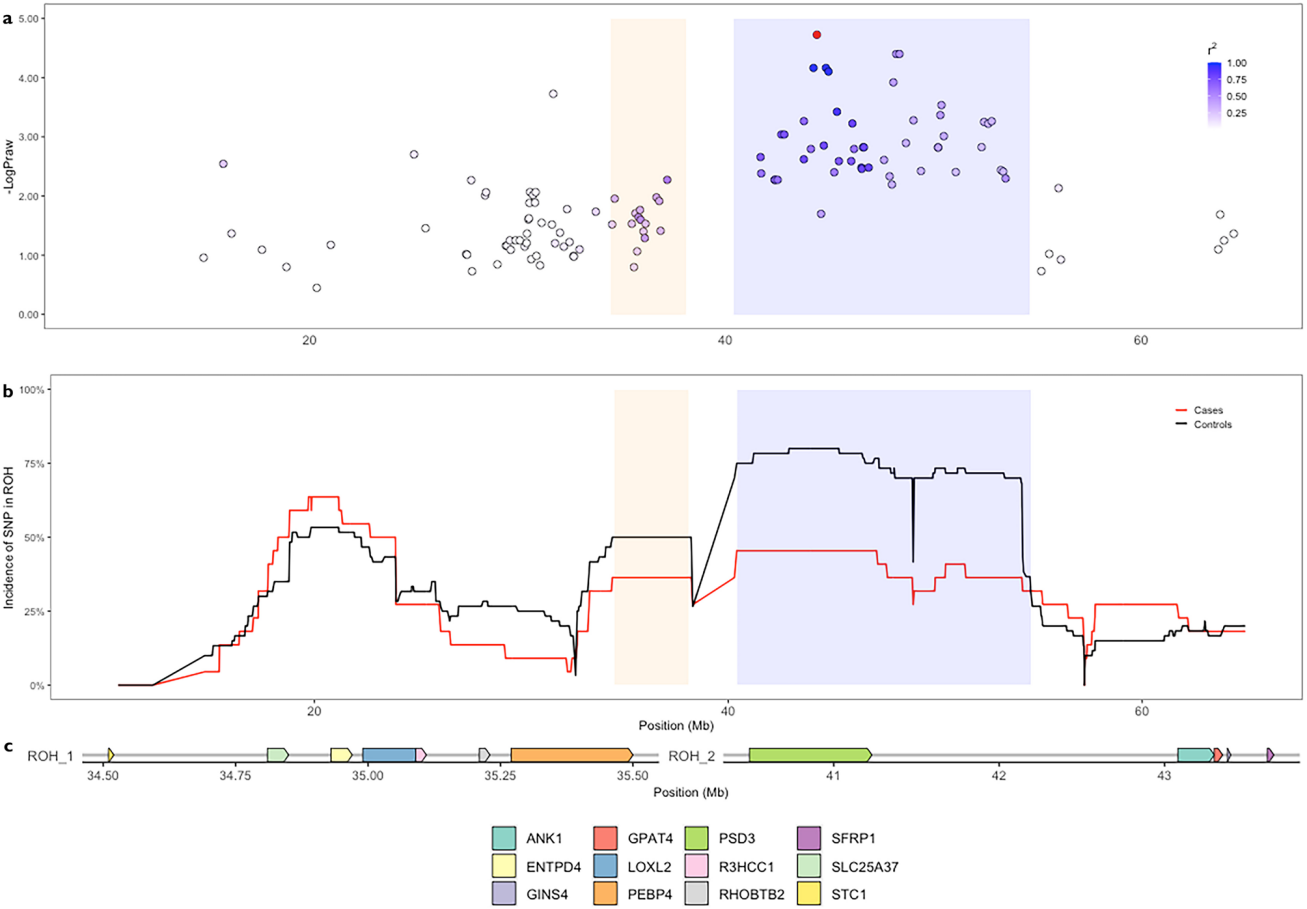
Overlapping FDM associated loci on chromosome B1 was identified in both ROH and GWAS analyses. (**a**) The distribution of SNVs flanking the highest associated SNV (red). Haplotype testing narrowed this to a region spanning 42–49 Mb. (**b**) Two ROH were observed on chromosome B1. ROH_1 (orange) was 4 Mb downstream of the GWAS identified FDM-association loci. ROH_2 (blue) was found to overlap the FDM-associated haplotype block. The incidence of SNVs included in ROHs were observed differentially between cases and controls with two adjacent peaks of higher frequently in controls than cases. **c.** Targeted variant discovery in these ROH and GWAS-identified regions revealed coding variants in genes.

Two ROH flanking the centromere on chromosome B1 were detected across all populations. Both were syntenic to a region on human chromosome 8 and overlapped the putative ticked coat phenotype locus in cats (OMIA 001484-9685). The first spanned chrB1:34,395,302–38,202,712 at a lower incidence (0.49) than the second, chrB1:40,408,022–54,187,390 bp (0.69). Missense and synonymous risk variants in Lysyl Oxidase homolog 2 *(LOXL2)* and Phosphatidylethanolamine-Binding Protein 4 *(PEBP4)* genes segregated in FDM-cases (Table 2). SNVs within the chrB1:34,395,302–38,202,712 bp ROH were in moderate LD (r^2^ = 0.5) to the highest associated SNV in the chrB1:40,408,022–54,187,390 bp ROH (Fig. 4a). ChrB1:40,408,022–54,187,390 overlapped the most significant region of FDM-association identified in analysis 2 (Fig. 4b) and was observed in 80% of controls and 49% in cases and contained 65 genes. FDM-risk variants segregating in cases could not be detected as only one diabetic WGS sample had the chrB1:40408022-54187390 ROH. A homozygous missense variant (c.163A > G) in exon 1 of Epoxide hydrolase 2 (*EPHX2)* was fixed across all WGS samples. Annotated FDM-risk variants discovered across ROH are presented in table S4.

## Discussion

We sought to identify novel genetic loci associated with FDM in the Australian Burmese population. GWAS exploits the non-random coinheritance of genetic variants (LD) to assay thousands of markers for an association with any phenotypic trait. The Burmese breed is characterised by a low fraction of GWAS-informative SNVs on the 63 K array, and only 49% of the available array markers were included in our analysis. However, the low allele frequency spectrum used in array-based association analyses, coupled with the genetic profile of this breed limits the need to control for false-positives. Given the extent of LD observed in pedigreed cat breeds, traditionally conservative forms of correction (i.e. Bonferroni correction) are considered excessive. Empirical estimations of genome-wide significance (i.e. 95% CIs from the distribution of raw P values) are more conservative than what is typically used in human GWAS but are adequate in population isolates^16,36^.

Genomic clustering of samples was concordant with geographical provenance. Geographic population stratification was expected as the cats in this study were collected from British, European and Australian populations that have each been subjected to population bottlenecks during their local breed development. Population substructure in GWAS cohorts is identified as a cause of potentially spurious associations^37^. Imprecise modelling of genetic relatedness within GWAS sample populations can cause substantial inflation of test statistics and potentially false association signals. Further, limiting study samples to entirely unrelated individuals is difficult in pedigreed breeds, such as the Burmese. To limit the effects of hidden relatedness and population stratification, the linear mixed-model (LMM) approach implemented by EMMAX was used. LMM have been shown to perform well in comparison to traditional family-based association tests^38^.

The Burmese cats in this study displayed high homozygosity and long ROH consistent with previous reports^14^. The distribution of ROH across the Burmese samples was consistent with previous results^39^. Long ROH (> 5 Mb) are indicative of recent inbreeding, unbroken by historical recombination events. No detectable difference in inbreeding coefficients between FDM cases and control groups was identified. Several factors influence the resolution of ROH calling, including marker density, marker distribution and genotype call quality. Therefore, for most populations, medium density genotyping arrays do not lend themselves to high resolution analysis of ROH^40^. For this analysis, minor allele frequency (MAF) and LD pruning were not employed in the ROH detection as both have been shown to mask the detection of true ROH by limiting genome coverage^39^. Long ROH (1–10 Mb) made up the largest size category observed across all populations, accounting for over 70% of ROH observations. Large ROH may persist in a population because of low rates of local recombination particularly in genomic regions that are subject to positive selection pressures. The risk of T2D is increased by moderate inbreeding and consanguinity within isolated populations^41–43^. ROH did not implicate autozygosity as a risk factor for FDM in Burmese cats, as diabetic cats did not display a higher number of ROH or higher inbreeding coefficient than non-diabetic cats.

Selective sweeps, indicative of positive selection, manifest by the reduction of genetic variation surrounding a beneficial mutation, and occur due to positive selection pressure increasing the frequency of the favourable allele over time^44^. In cats, at least eight loci are involved in coat colour determination, with various combinations of these responsible for extensive phenotypic variation. Genes, *TYR* and *TYRP1*, inside ROH support strong selection of tyrosine metabolism and common Burmese coat ‘ticking’ and colour phenotypes^45–47^. Tyrosine metabolism influences the development, differentiation and proliferation of melanocytes, the construction and transport of the melanosome and the synthesis of melanin^48^. Short selective sweeps, indicative of extensive LD, have been associated with T2D in isolated human populations^49^. Sweeps can cause a shift in the allelic frequency of a selected allele and the ‘hitchhiking’ alleles in their vicinity. Pancreatic islets of FDM and T2D patients are characterised by reduced beta cell function and decreased insulin gene expression^50,51^. FDM-risk variants within these selective sweeps identified *GRM5* and *DLG2* as candidates for beta cell dysregulation, as both are involved in glucose-stimulated insulin secretion and hyperglycaemia susceptibility in rodent and human^52–54^. Additionally, *PTPRD* contained within the chromosome D4 ROH has been associated with progression to diabetes in humans through enhanced insulin resistance^55,56^. The ROH on chromosome B3 overlapped a syntenic loci in humans implicated in Prader Willi Syndrome (PWS). T2D has been found to affect between 7 and 24% of PWS patients, far exceeding the prevalence in the general population^57,58^. This is likely a consequence of insulin resistance resulting from morbid obesity however PWS patients also exhibit a state of hypoinsulinaemia without expected insulin resistance despite their obese state. Deficits in pancreatic islet development may play a role in the PWS phenotype^59,60^. *NIPA1* is a magnesium transporter, upregulated in response to reduced magnesium concentration^61^. Variants in *NIPA1* have previously been associated with T2D risk^62^ and *GABRG3* is an early childhood obesity gene contributing to PWS phenotype^63^. The basis of the diabetic state in obese PWS patients is currently unclear but the PWS locus contains epigenetically imprinted genes that serve as viable candidates for FDM.

The most compelling FDM-association was provided by the locus on chromosome B1, identified in both ROH and GWAS analyses. Coding variants matching the risk-haplotype segregated in *ANK1*, an established T2D candidate gene associated with decreased beta-cell function in humans^64,65^. Feline *ANK1* is highly orthologous (93.89%) to human *ANK1* sequence and multiple *ANK1* isoforms with affinities for various target proteins are expressed in a tissue specific manner^66–68^. Transcripts of varying size are present in tissues essential to glucose metabolism: skeletal muscle^69^, pancreas, adipose and liver^70^. Splice region variants identified in this study may be influential in the molecular function of feline *ANK1* isoforms, but any potential impact of these will be dependent on tissue-specific expression of *ANK1* isoforms. Alternatively-spliced *ANK1* transcripts are functionally diverse and variants in regulatory regions have been implicated in altered molecular function of the various human isoforms and their role in T2D^71^. The physiological role of *ANK1* in T2D pathogenesis is unconfirmed but *Ankyrin B* (ANK2) regulates ATP sensitivity in murine pancreatic beta cells^72^. Additionally, SNVs in the *ANK1* promoter region have been found to increase intramuscular fat content in pigs^73^ and increased muscle-specific *ANK1* expression in human skeletal muscle^71^ implying SNVs in the *ANK1* promoter region may play a role in the development of insulin resistance.

ROH analysis implicated two loci on chromosome B1 as FDM-risk loci, containing risk variants segregating in cases and across the breed, further highlighting the genetic complexity of FDM. Both partially overlapped the semi-dominant Abyssinian ‘ticked’ coat locus responsible for the homogenous agouti coat with no body markings characteristic of Burmese cats^47^. Across chrB1:34,395,302–38,202,712, variants segregated in cases in *LOXL2*, previously implicated in nephropathy and retinopathy in T2D patients^74,75^, indicating these may play a critical role in the progression to FDM. Diabetic nephropathy is not routinely recognised in diabetic cats, although diabetic retinopathy has been reported^76^. Dyslipidaemia is a critical factor in the early inflammatory response in the development of retinopathy^77^. *LOX2* overexpression is considered a potential target for treatment of vascular changes involved in diabetic retinopathy^78^.

Inherited derangements in lipid metabolism have been described in the Burmese breed^20,79^ and may be related to the high prevalence of FDM. Familial hypercholesterolaemia (FH) is a common autosomal dominant disorder of lipoprotein metabolism, with variable phenotypic presentation in humans^80^. Variants in *EPHX2* have been found to exacerbate the dysfunction of the *Low-density lipoprotein receptor* (*LDLR*) gene in FH^81^ and are associated with insulin resistance in T2D patients^82^. Defects in *LDLR* result in disturbed clearance of low-density lipoprotein cholesterol (LDL-C) and the clinical presentation varies widely depending on the segregation of risk alleles and haplotype. A missense substitution (c.163A > G) in *EPHX2* was fixed across all WGS Burmese samples within the chrB1:40,408,022–54,187,390 bp ROH. Phenotypic heterogeneity is present between homozygous and heterozygous FH patients, with a range of increased plasma triglyceride levels, likely influenced by modifying gene–gene interactions. A syndrome similar to FH has been described in Burmese cats with affected individuals exhibiting significantly elevated plasma triglyceride concentrations, lipid aqueous and delayed triglyceride clearance compared with other breeds^83–85^. Post-prandial hypertriglyceridemia has been described in cats with inherited *Lipoprotein lipase (LPL)* factors^86^ with variable phenotypic presentation in homozygous and heterozygous individuals. Further studies will be required to determine whether the lipid metabolism defects in Burmese cats can be attributed to *EPHX2* dysfunction and any potential contribution to increased risk of FDM.

This study has unveiled genomic regions underlying dysfunctional metabolic processes predisposing Burmese cats to FDM, highlighting lipid metabolism as a contributing factor. Larger cohorts of comprehensively phenotyped individuals across multiple breeds are needed to validate the genetic risk factors presented here. The segregation of polymorphisms across autozygous regions and identification of risk-haplotypes reveal a potential highly penetrant recessive locus on chromosome B1. This strategy for identifying risk-loci across a predisposed population suggests the interaction of multiple genes across a fixed number of loci are likely responsible for FDM in Australian Burmese cats. The regions reported here characterise a window of FDM association containing candidate genes for further investigation in larger studies with prospective clinical phenotyping. Detailed characterisation of the genetic risk factors involved in the pathogenesis of FDM, including the allelic variants and candidate genes presented here, will provide a more comprehensive understanding of the molecular mechanisms involved and the genetic interaction profiles responsible for this disease, and for T2D in humans. Building on insights gained from this study, future work can potentially pinpoint population specific allele frequency profiles.

## Materials and methods

### Clinical diagnosis and sample collection

Eighty-two Burmese cats, comprising 22 diabetic and 60 non-diabetic individuals were submitted for genotyping analysis on the Illumina Infinium iSelect 63 k Cat DNA genotyping array. Genomic DNA was extracted from whole blood using the DNeasy blood and tissue kit (Qiagen GmbH, Germany) or Performagene PG-100 buccal swabs (DNA Genotek, Canada). All cases (15 male, 7 female) had been diagnosed with FDM based on persistent fasting hyperglycaemia, glycosuria with clinical signs; weight loss, polydipsia and polyphagia by a qualified veterinarian at the University of Sydney Veterinary Teaching Hospital and Animal Diabetes Australia. Unaffected Burmese were collected from an database of adult Burmese cats. This included 23 Australian and 24 British Burmese and 13 European Burmese, nine European cats were members of the same pedigree^19^. Samples from non-diabetic Burmese (26 male, 34 female) were all collected by a qualified veterinarian and showed no clinical signs of FDM at the time of their presentation for clinical examination and sample collection and had never previously been diagnosed with FDM. Veterinary records of controls were checked for indicators of disease related to diabetes before they were included.

### Remapping array variants to feline 9.0 genome assembly

To determine the exact position of feline array single nucleotide variants (SNV) on the Felis_catus_9.0 genome assembly, we performed a Basic Local Alignment Search^87^ using SNV probes available in the feline manifest^88^. A FASTA file was created using the SNV identifier and flanking genomic sequence upstream and downstream of each SNV in top orientation. The longest flanking sequence was retained and output in FASTA format. A custom BLAST database was prepared using BLAST+^87^ to make the reference FASTA sequence searchable. A nucleotide BLAST search was performed and output was filtered to collect the single best hit for each sequence, hits that failed to return any result, hits that failed to reach the base position immediately adjacent to the SNV and those that had an E-value greater than 1e−05 were rejected.

### Population structure and quality control

Quality control was carried out for 82 samples and 61,386 SNVs using PLINK 1.9^89^. First, we identified possible duplicate samples and outliers based on pairwise genetic distances (–genome) and removed one sample from each pair with an identity by descent (IBD) estimate > 0.65. Population stratification was evaluated using a MDS plot with two dimensions (–mds). Marker-based QC included pruning the total set of SNVs at a MAF (–maf) of 0.05 and a SNV (–mind) and individual call rate (–geno) of > 90%.

### Case–control genome-wide association study and haplotype analysis

Testing for association between FDM and markers on the feline genotyping array was performed in two stages using Efficient Mixed-Model Association eXpedited (EMMAX)^90^. Given all cases were of Australian provenance, an initial association analysis (analysis 1) was performed on samples within the unstratified Australian cluster. To validate the associations observed in the Australian cluster, a second association analysis (analysis 2) run in EMMAX included an expanded group of individuals from Australian, UK and EU populations (22 cases, 60 controls) and the top 10% of association signals from analysis 1. The genome-wide significance threshold in both analyses was calculated based on empirical 95% confidence intervals (CIs). The probability distribution was determined by running the GWAS 1000 times with randomly permuted phenotypes. The genome-wide significance threshold was set at the 97.5% upper CI (based on a two-tailed distribution) (*P* < 7.6 × 10^−5^) (Karlsson et al.^16^).

Regions of association were refined using LD clumping (–clump) in PLINK. First, a region of weak LD (r^2^ > 0.2) was defined within 5 Mb of each top SNV and then the region was narrowed to a single locus of high LD (r^2^ > 0.8) within 2 Mb of the highest associated SNV per locus. The refined regions were submitted for haplotype analysis with HAPLOVIEW^91^. FDM-associated haplotype blocks were examined in all 82 individuals and were defined using the four-gamete rule^92^. Consecutive haplotype blocks with a multiallelic r^2^ value of 1 were combined and blocks containing the top associated SNVs were used to define our FDM-associated loci. Significance of FDM-associated haplotype blocks was measured by running 10,000 permutations.

### Runs of homozygosity analysis

ROH analysis was performed to identify FDM-associated regions of autozygosity using PLINK (–homozyg function). The input settings for minimal density of SNVs, maximal gap size, scanning window length and threshold settings were determined empirically^39^. The ROH analysis was run using a maximal gap size of 300 kb (–homozyg-gap) and scanning window size setting of minimal density (–homozyg-density) at 80 kb/SNV at a genome coverage of 98.9%. Additional settings were; a scanning window hit rate of 0.05 (–homozyg-window-threshold), maximum of one heterozygous SNV per final ROH segment (–homozyg-het) and a minimum of 94 SNVs in each final ROH (–homozyg-SNV) (Table S2). Inbreeding coefficients (F_ROH_) for genotyped individuals were calculated based on the length of the autosomal genome and the length of the genome covered by feline array markers (Meyermans et al. 2019). Correlations between inbreeding coefficients F_ROHcov_ and F_ROHaut_ were measured using a Pearson’s correlation test. We did not prune SNVs based on MAF or LD. ROH on chromosomes B1, B3, D1 and D4 were examined for syntenic regions using the Ensembl Bioinformatics database and a database search was performed using the BLAST algorithm. Comparative genome analyses of ROH on chromosomes B1 and B3 were undertaken. ROH distributions were compared between the Australian cluster and the expanded group of Burmese and between cases and controls across all autosomes to identify any regions associated with FDM.

### Risk variant discovery and annotation

Genomic DNA was extracted from whole blood samples of five Australian Burmese cats (3 cases, 2 controls) included in GWAS analysis, using a phenol–chloroform extraction protocol. DNA samples were submitted for whole genome sequencing (WGS). Illumina paired-end libraries were prepared and sequenced on the Illumina HiSeq 2000 platform with 150 bp paired-end reads (~ 12–21 × coverage). An additional three WGS samples, of unknown diabetic phenotype, available on Sequence Read Archive (SRA) were downloaded (SRX2376210, SRX2669131, SRX2376201) (~ 12–27 × coverage). All samples were aligned to the Felis_catus_9.0 reference assembly using BWA-mem^93^. Base quality score recalibration, indel realignment and duplicate removal was performed using Genome Analysis ToolKit (GATK)^94^. Genome-wide variant detection was performed using GATK’s HaplotypeCaller according to best practices and variants filtered for sequence depth (DP > 10), quality of alignment (GQ > 20) and strand bias^95^. Given the complex phenotypic presentation of FDM and the unknown clinical phenotype of the SRA samples, associated haplotype blocks were filtered according to presence or absence of risk haplotypes across the eight WGS samples, rather than clinical information of each individual. ROH were examined for FDM risk variants segregating in affected WGS samples and potential risk factors across the breed. Filtered variants were analysed with Ensembl’s Variant Effect Predictor (VEP) tool^96^. Sequence variants were further filtered for genic variants and annotated variants with Sort Intolerant From Tolerant (SIFT) tool^97^.

### Ethical approval

Recommendations from the Australian Code for the Care and Use of Animals for Scientific Purposes was strictly adhered to throughout this study. Research was conducted at The University of Sydney, under Animal Ethics Committee approval no: N00/9–2009/3/5109, 24 September 2009. Blood and buccal swab samples were all collected a veterinarian or donated by owners and breeders.

## Supplementary information

Supplementary Information.

## Data Availability

Genotyping array data is available at 10.6084/m9.figshare.12561815 and 10.6084/m9.figshare.12561770. Whole genome sequence data for five phenotyped Australian Burmese samples can be accessed freely upon request to the 99Lives Consortium Coordinator, L. A. Lyons (lyonsla@missouri.edu). Whole genome sequence data for three Burmese samples of unknown clinical status can be accessed via NCBI Sequence Read Archive under accession codes: SRX2376208, SRX2376209 and SRX2376210.
