# Author Correction: Mapping the genetic basis of diabetes mellitus in the Australian Burmese cat (*Felis catus*)

**DOI:** 10.1038/s41598-021-83769-x

**Published:** 2021-02-17

**Authors:** Georgina Samaha, Claire M. Wade, Julia Beatty, Leslie A. Lyons, Linda M. Fleeman, Bianca Haase

**Affiliations:** 1 Faculty of Science, Sydney School of Veterinary Science, University of Sydney, Sydney, NSW Australia; 2 School of Life and Environmental Sciences, The University of Sydney, Sydney, NSW Australia; 3 Department of Infectious Diseases and Public Health, City University of Hong Kong, Kowloon, Hong Kong SAR People’s Republic of China; 4 Department of Veterinary Medicine and Surgery, College of Veterinary Medicine, University of Missouri, Columbia, MO USA; 5 Animal Diabetes Australia, Melbourne, VIC Australia

Correction to: *Scientific Reports* 10.1038/s41598-020-76166-3, published online 05 November 2020

This Article contains typographical errors.

In the Abstract,

“The locus on chromosome B1, common to both analyses, revealed coding and splice region variants in candidate genes, *ANK1, EPHX2* and *LOX2,* implicated in diabetes mellitus and lipid dysregulation.”

now reads:

“The locus on chromosome B1, common to both analyses, revealed coding and splice region variants in candidate genes, *ANK1, EPHX2* and *LOXL2,* implicated in diabetes mellitus and lipid dysregulation.”

In the Discussion,

*“LOX2* overexpression is considered a potential target for treatment of vascular changes involved in diabetic retinopathy^78^.”

now reads

*“LOXL2* overexpression is considered a potential target for treatment of vascular changes involved in diabetic retinopathy^78^.”

This has now been corrected in the PDF and HTML versions of the Article.

